# Frequency of IFNγ-producing T cells correlates with seroreactivity and activated T cells during canine *Trypanosoma cruzi* infection

**DOI:** 10.1186/1297-9716-45-6

**Published:** 2014-01-23

**Authors:** Ashley N Hartley, Gretchen Cooley, Sarah Gwyn, Marcela M Orozco, Rick L Tarleton

**Affiliations:** 1 Center for Tropical and Emerging Global Diseases, University of Georgia, Athens, GA 30602, USA; 2 Department of Infectious Diseases, University of Georgia, Athens, GA 30602, USA; 3 Departamento de Ecología, Laboratori de Eco-Epidemiología, Genética y Evolución, Facultad de Ciencias Exactas y Naturales, Universidad de Buenos Aires, Ciudad Autónoma de Buenos Aires, Argentina; 4 Department of Cellular Biology, University of Georgia, Athens, GA 30602, USA

## Abstract

Vaccines to prevent *Trypanosoma cruzi* infection in humans or animals are not available, and in many settings, dogs are an important source of domestic infection for the insect vector. Identification of infected canines is crucial for evaluating peridomestic transmission dynamics and parasite control strategies. As immune control of *T. cruzi* infection is dependent on humoral and cell-mediated immune responses, we aimed to define a serodiagnostic assay and T cell phenotypic markers for identifying infected dogs and studying the canine *T. cruzi*-specific immune response. Plasma samples and peripheral blood mononuclear cells (PBMCs) were obtained from forty-two dogs living in a *T. cruzi*-endemic region. Twenty dogs were known to be seropositive and nine seronegative by conventional serologic tests two years prior to our study. To determine canine seroreactivity, we tested sera or plasma samples in a multiplex bead array against eleven recombinant *T. cruzi* proteins. Ninety-four percent (17/18) of dogs positive by multiplex serology were initially positive by conventional serology. The frequency of IFNγ-producing cells in PBMCs responding to *T. cruz*i correlated to serological status, identifying 95% of multiplex seropositive dogs. Intracellular staining identified CD4^+^ and CD8^+^ T cell populations as the sources of *T. cruzi* lysate-induced IFNγ. Low expression of CCR7 and CD62L on CD4^+^ and CD8^+^ T cells suggested a predominance of effector/effector memory T cells in seropositive canines. These results are the first, to our knowledge, to correlate *T. cruzi*-specific antibody responses with T cell responses in naturally infected dogs and validate these methods for identifying dogs exposed to *T. cruzi*.

## Introduction

*Trypanosoma cruzi* infects humans, wildlife, and domestic animals throughout the Americas. Worldwide, it is estimated that at least ten million individuals are chronically infected with *T. cruzi*, with twenty-five million more are at risk of infection [1]. *T. cruzi* infection predominantly burdens countries of Central and South America but encompasses all of the Americas including the United States, contributing 14 000 deaths annually and 700 000 daily adjusted life years [2]. Current methods of parasite control, diagnostics, and treatment are inadequate in completely disrupting transmission as new infections occur annually, and no vaccines are currently available for human or veterinary use.

Dogs play a significant role in *T. cruzi* epidemiology and ecology as reservoirs for infection [3]. Preferential [4] and host tolerant [5] vectorial feeding of dogs, higher *T. cruzi* infectivity of *Triatoma infestans* upon feeding on dogs compared to human feeding [6], and the close proximity of dogs to humans in domiciles in *T. cruzi* endemic regions [7] identifies dogs as a critical control point for *T. cruzi* transmission. Strategies for interrupting transmission by targeting the dog have included insecticide-impregnated dog collars [8] and various means of vaccination [9-11]. Despite these efforts, peridomestic *T. cruzi* transmission still occurs between humans, dogs, and the insect vector [3]. Identifying infected canines and understanding the immune mechanisms responsible for canine *T. cruzi* recognition and control are critical for designing and evaluating future intervention strategies targeting canines.

Because the results of individual serological tests for *T. cruzi* infection are not considered to be definitive, positive responses on a minimum of two tests is generally recommended to identify infected humans [12-14], and dogs [15,16]. For identifying circulating anti-*T. cruzi* antibodies, hemagglutination, complement fixation, indirect immunofluorescence, and direct agglutination tests have been standardized for canine sera [17]. These methods, also known broadly as conventional serological assays, predominantly utilize insect stage epimastigote-derived antigens for seroreactivity testing. In an attempt to improve the quality of serological tests for detection of *T. cruzi* infection, we developed a multiplex bead array format utilizing recombinant *T. cruzi* proteins selected for their predicted expression during mammalian infection stages [18], high abundance in trypomastigote and amastigote proteomes [19], and ability to detect the broad array of responses observed in *T. cruzi*-infected humans [20]. Investigating serological assays employing mammalian-stage derived proteins could maximize discrimination of seropositive and seronegative canines, thereby reducing the need for multiple tests to identify seroreactive dogs during intervention campaigns.

Studies of human *T. cruzi* infection and experimental animal models have highlighted the role antibodies [21-23], CD4^+^ and CD8^+^ T cells [24-26], and effector cytokines [27,28] serve in immune control of *T. cruzi*, predicting disease development, and determining treatment success. Identification of *T. cruzi*-specific CD8^+^ T cells by class I MHC tetramers for mice [29] and humans [27] has facilitated monitoring of cell surface marker expression and effector function of *T. cruzi*-specific T cells. In particular, correlation of parasitological cure with increased CD127 and CD62L expression by *T. cruzi*-specific CD8^+^ T cells in benznidazole-treated mice has identified biomarkers for evaluating parasite burden in vivo [30]. Characterizing similar canine T cell phenotypes and effector cytokine production is critical for understanding the development of an appropriate *T. cruzi*-specific immune response in the dog and evaluating modulation of this response following intervention strategies targeting the dog.

The aim of this study was to test the use of a multiplex serodiagnostic assay for *T. cruzi* in dog sera or plasma and to further evaluate T cell phenotypes and effector cytokine production associated with canine *T. cruzi* infection. Peripheral blood was collected from dogs previously tested by conventional serology and/or xenodiagnosis and living in a *T. cruzi*-endemic region of northern Argentina. Plasma were submitted to multiplex bead array analysis using eleven recombinant *T. cruzi* proteins, previously described for identifying seroreactive humans and evaluating treatment success [20], and PBMCs were assessed for IFNγ production in response to *T. cruzi* amastigote antigens by ELISpot. Utilizing recently identified T cell reagents identified for the dog (Hartley and Tarleton, unpublished), we assessed expression of canine T cell surface markers associated with naïve or central memory and activated T cells. The results of this study determined dogs living in *T. cruzi*-endemic regions develop robust anti-*T. cruzi* antibody responses which correlate with T cell effector and activation phenotypes. The serological and T cells assays described here provide a platform for monitoring canine immune responses and for developing and evaluating canine-centric *T. cruzi* intervention strategies.

## Materials and methods

### Animals

A maximum of 10 mL of blood from 42 dogs living in four villages in Pampa del Indio, Chaco, Argentina were drawn into heparinized tubes (BD Vacutainer, BD, Franklin Lakes, NJ, USA) by venipuncture. A proportion of these dogs had been previously screened and found to be seropositive or seronegative for *T. cruzi* by conventional serology (see below). Approximately 50 mL of blood from three clinically healthy dogs and sera from 5 additional healthy dogs from the United States were obtained to serve as controls. Isolation of peripheral blood mononuclear cells (PBMCs) occurred within 24 h of collection. Briefly, PBMCs were isolated by density gradient centrifugation on Lymphocyte Separation Medium (MP Biomedicals, Solon, OH, USA), resuspended in complete RPMI 1640 supplemented with 10% heat-inactivated FCS (HyClone Laboratories, ThermoScientific, Logan, UT, USA), and stored frozen at -80 °C. These purification, storage, and recovery procedures yielded > 80% viability for Argentinean and > 95% viability for North American dogs as determined by microscopic examination of trypan blue exclusion. Plasma samples were collected during PBMC isolation and stored frozen at -20 °C. Informed oral consent was requested and obtained from each head of household for samples collected in Argentina. Animal use protocols for samples collected in the United States were approved by the University of Georgia Institutional Animal Care and Use Committee. Blood collection in Argentina was conducted according to the protocol approved by the Argentinean “Dr Carlos Barclay” Independent Ethical Committee for Clinical Research (IRB No. 00001678, NIH registered, and Protocol No. TW-01-004).

### Initial conventional serology and xenodiagnosis

Indirect hemagluttination assay (IHA) and an in-house enzyme-linked immunosorbent assay (ELISA) modified from a standardized protocol [15] were used to test sera for anti-*T. cruzi* antibodies two years prior to collection of PBMC and plasma for this study [31]. Sera were considered discordant if results of IHA and ELISA reactivity mismatched. A subset of animals were also examined by xenodiagnosis, as previously outlined [31] and notated in Table 1. Briefly, boxes containing uninfected fourth-instar nymphs of *T. infestans* were exposed to the abdomen of an individual dog. Insect feces were examined microscopically at 30 and 60 days later for *T. cruzi* infection.

**Table 1:** **Comparison of xenodiagnosis**, **initial conventional serology**, **multiplex serology**, **and IFNγ ELISpot assays**

**Dog ID#**	**Xenodiagnosis**	**Initial serology**	**Multiplex serology**	**IFNγ ELISpot SFU**
Non-responders				
160	-	-	0	0
163	-	-	0	0
280	-	-	0	0
281	-	-	0	0
283	-	-	0	3
187	n.d.	-	0	0
258	n.d.	-	0	0
259	n.d.	-	0	0
267	n.d.	-	0	0
198	n.d.	+	0	0
200	n.d.	+	0	0
588	n.d.	discordant	1	0
260	n.d.	n.d.	0	0
B	n.d.	n.d.	0	0
D	n.d.	n.d.	0	0
F	n.d.	n.d.	0	0
I	n.d.	n.d.	0	n.d.
J	n.d.	n.d.	0	0
K	n.d.	n.d.	0	3
L	n.d.	n.d.	0	0
M	n.d.	n.d.	0	0
			**0**/**21**	**0**/**20**
Responders				
152	-	+	6	400
71	+	+	5	55
154	+	+	3	108
157	+	+	5	13 (-)
159	+	+	3	290
266	+	n.d.	3	55
272	+	+	2	50
274	+	+	2	68
276	+	+	4	363
284	+	+	3	955
43	n.d.	+	4	233
67	n.d.	+	1	80
69	n.d.	+	5	550
193	n.d.	+	4	130
197	n.d.	+	2	63
434	n.d.	+	5	58
572	n.d.	+	4	n.d.
573	n.d.	+	3	398
589	n.d.	+	4	33
P	n.d.	n.d.	3	823
O	n.d.	n.d.	5	520
			**20**/**21**	**19**/**20**
Controls (US)				
1	n.d.	n.d.	0	0
2	n.d.	n.d.	0	3
3	n.d.	n.d.	0	0
4	n.d.	n.d.	0	n.d.
5	n.d.	n.d.	0	n.d.
6	n.d.	n.d.	0	n.d.
7	n.d.	n.d.	0	n.d.
8	n.d.	n.d.	0	n.d.
			**0**/**8**	**0**/**3**

Forty-two samples from dogs living in a *T. cruzi*-endemic region are grouped as non-responders and responders based on reactivity to *T. cruzi* lysate in serological and effector function assays. Test reactivity is annotated as positive (+), negative (-), discordant (disc), or not determined (n.d.). Controls are canine samples from dogs living in the United States. Bold data are results presented as # positive individuals/# total individuals of the groups.

### *T. cruzi* amastigote lysate

Brazil strain trypomastigotes were cultured overnight in pH 5 RPMI 1640 supplemented with 10% FCS and 10 mM phosphate citrate buffer to transform trypomastigotes into amastigotes. After two PBS washes, parasites were frozen at -20 °C. Frozen aliquots were subjected to five freeze/thaw cycles followed by three 10s sonications in a FS15 sonicator (Fisher Scientific, Pittsburgh, PA, USA). The supernatant of a 12 000 rpm centrifugation was collected, filter sterilized, and protein concentration determined by Bradford assay.

### Serology testing with protein multiplex bioassay

Canine plasma samples were tested with a recombinant *T. cruzi* protein multiplex assay previously described in detail for testing *T. cruzi* reactivity of human sera samples [20]. Briefly, sera or plasma are diluted 1:500 and incubated with a pool of 11 recombinant proteins attached to addressable Liquichip Ni-NTA beads (Qiagen Inc, Valencia, CA, USA) and *T. cruzi* amastigote lysate coupled to Carboxy-Ni-NTA beads (Qiagen Inc). Following washing, antibody binding was detected with goat anti-dog IgG conjugated to phycoerythrin (Santa Cruz Biotechnology Inc, Santa Cruz, CA, USA) and quantified on a Bio-Plex Suspension Array System (Bio-Rad, Hercules, CA, USA). Serum samples were assayed in duplicate and weighted mean fluorescence intensity (MFI) was calculated. The ratio of the specific MFI for each antigen versus a negative control (green fluorescent protein) protein was calculated for each antigen and sera or plasma in the assay.

### IFNγ ELISpot assays

Four hundred thousand PBMCs were cultured in media, 2 ng/mL phorbol 12-myristate 13-acetate (PMA) and 500 ng/mL Ca^2+^ ionomycin (both Sigma-Aldrich, St. Louis, MO, USA), or 10 μg/mL *T. cruzi* amastigote lysate for 16 h at 37 °C and 5% CO_2_. Cells were assayed for IFNγ production using the Canine IFN-γ ELISpot kit (cat no. EL781, R& D Systems, Inc, Minneapolis, MN, USA) following standard kit protocol. Enumeration of spot forming cells was completed by an ImmunoSpot analyzer (CTL, Cleveland, OH, USA). Mean numbers of spots from duplicate or triplicate well were obtained for media and *T. cruzi* lysate stimulation conditions. Responses were considered positive if a minimum of 20 spots/10^6^ PBMC total were present and this number was at least twice the value of wells assayed with media alone [27].

### Intracellular cytokine staining

For assaying IFNγ production, 4 × 10^5^ PBMCs were stimulated for 5 h in the presence of 2 ng/mL PMA, 4 μg/mL Ca^2+^ ionomycin (Sigma-Aldrich), and brefeldin A (BD GolgiPlug; BD Biosciences, San Jose, CA, USA) adapted from described stimulation conditions [32] or overnight with 40 μg/mL *T. cruzi* lysate at 37 °C. Brefeldin A was added 5 h prior to end of incubation, and the cells were stained with anti-CD8-Pacific Blue and anti-CD4-FITC (AbD Serotec, Raleigh, NC, USA) followed by intracellular staining with anti-bovine IFN-γ AF647 (AbD Serotec) according to the BD Cytofix/Cytoperm kit (BD Biosciences). Samples were fixed in 2% formaldehyde prior to analysis by flow cytometry.

### CTL2.58 purification and PE-Cy7 labeling

Culture supernatants containing clone CTL2.58 IgG were a kind gift of Dr Mary Tompkins, North Carolina State University, Raleigh, NC. Immunoglobulins were purified according to instructions of the Pierce Thiophilic Adsorption Kit (cat no 44916, Thermo Scientific, Rockford, IL, USA). Elutions containing targeted heavy and light chain were confirmed by SDS-PAGE and conjugated to PE-Cy7 using Lightning-Link PE-Cy7 antibody labeling kit (cat no 762-0015, Novus Biologicals, Littleton, CO, USA) following manufacture instructions. ConA-stimulated canine PBMCs were used for positive reactivity for CTL2.58-Pe-Cy7 staining (Additional file 1).

### Cell surface phenotyping

PBMCs (500 000) were incubated in complete RPMI 1640 supplemented with 10% heat-inactivated FCS for 1 h at 37 °C, centrifuged, and resuspended in 100 μL of CCL19-hIg (ELC; [33]) culture supernatant for 45 min at 4 °C. After incubation, cells were washed and resuspended for staining in PAB solution containing PBS with 1% BSA and 0.05% sodium azide (both from Sigma-Aldrich). Antibodies used were anti-CD8 PacBlue, anti-CD4 AF647, anti-CD62L PE (AbD Serotec), CTL2.58-PeCy7, and anti-human IgG AF488 (Molecular Probes, Eugene, OR, USA). 7-amino-actinomycin D (7AAD, BD Pharmingen, BD Biosciences, San Jose, CA, USA) was included for live/dead cell discrimination. Following incubation of PBMCs with antibody mixes for 45 min on ice, cells were washed twice, fixed in 2% formaldehyde, and analyzed by flow cytometry.

### Flow cytometry and statistical analysis

Fixed PBMC samples were collected on a CyAn ADP using Summit, version 4.3 (Beckman Coulter, Fullerton, CA, USA). FlowJo flow cytometry analysis software, version 8 (Tree Star, Ashland, OR, USA) was used for analyses. Positive gates were determined by relevant fluorescence minus one controls. Statistical analyses were performed using Prism v4.0c (GraphPad Software, La Jolla, CA, USA).

## Results

**Figure 1:**
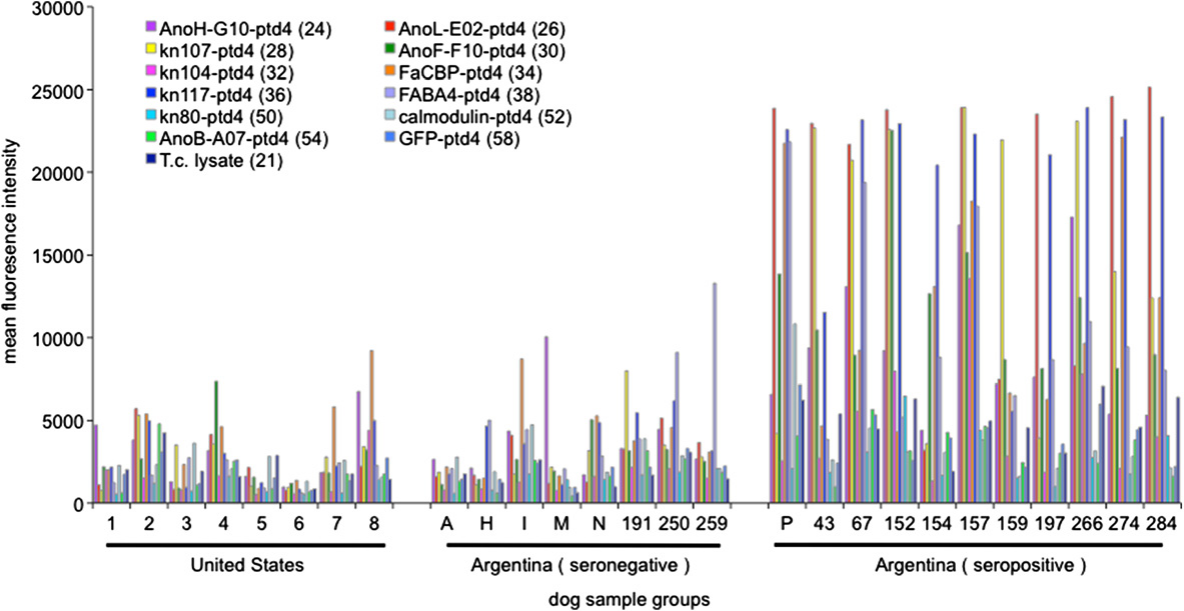
**Canine plasma samples tested with a multiplex bead array correlate with initial conventional serology results.** Reactivity to 11 *T. cruzi* recombinant proteins in sera or plasma from conventional serology positive (seropositive) and negative (seronegative) dogs living in a *T. cruzi*-endemic region of Argentina and canine sera obtained from dogs in the United States. Vertical bars represent mean fluorescence intensity (MFI) to the recombinant protein on a scale from 0 to 30 000 arbitrary light units. Recombinant GFP and *T. cruzi* lysate-coated beads served as negative and positive controls, respectively.

Our first aim was to test an improved serodiagnostic assay for the ability to detect *T. cruzi* infection in canines. Sera and plasma samples were obtained from eight healthy dogs from the United States (US) and forty-two dogs living in a *T. cruzi*-endemic region of northern Argentina. Twenty Argentinean dogs were known to be seropositive, nine seronegative, and one discordant by conventional serology two years prior to this study. Ten dogs were new additions to domiciles and had not been previously tested. We employed a multiplex bead array format previously utilized for identifying and discriminating humans reactive to recombinant *T. cruzi* proteins, in which proteins were selected for their predicted expression during mammalian infection stages and high abundance in the trypomastigote and amastigote *T. cruzi* proteome [20]. Addressable beads were bound to eleven recombinant *T. cruzi* proteins, incubated with sera or plasma, followed with a secondary fluorophore conjugated anti-canine specific IgG, and quantified on a BioPlex Suspension Array System (BioRad) for mean fluorescence intensity (MFI). We first tested a subset of dogs and found that as expected, animals positive by conventional serology had considerably higher MFI values than United States control dogs or seronegative Argentinean dogs (Figure 1). All proteins in the multiplex assay showed reactivity with serum from at least one dog with a subset of six recombinant proteins (60S acidic ribosomal subunit protein, microtubule-associated protein homolog, glycosomal phosphoenolpyruvate carboxylase, flagellar calcium binding protein, major paraflagellar rod protein, and a hypothetical protein) identifying all initial conventional serology positive canines. All conventional serology positive dogs showed reactivity above a MFI of 10 000 to at least one protein or greater than 6500 MFI for at least four proteins in the multiplex array. One discordant dog, dog 588, had no significant responses to the subset of six recombinant proteins identifying all conventional serology positive canines. However dog 588 did have greater than 10 000 MFI to calmodulin protein-coated beads, but this reactivity was not observed in any other dog. These results validate a multiplex serological assay platform for identifying circulating anti-*T. cruzi* antibodies in the dog using mammalian-stage derived *T. cruzi*-recombinant proteins.

We next tested all Argentinean canine plasma samples creating a metric of reactivity by calculating the standard deviation of each individual plasma sample for each individual protein over reactivity to all conventional serology negative animals. Using a minimum reactivity to two proteins with four standard deviations above that of the seronegative individuals, ninety-four percent (17/18) of the dogs positive by multiplex serology were initially positive with conventional serology (Table 1). Two conventional serology positive dogs (dogs 198 and 200) had no significant responses to any recombinant proteins in the multiplex assay. All nine initial seronegative dogs were also negative in the multiplex bead serological assay. Analysis of eleven dogs without previous conventional serology revealed two additional seropositive animals, dog O and P.

**Figure 2:**
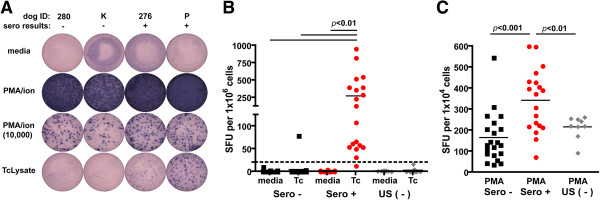
**Seropositive dogs have significantly higher frequencies of *T. cruzi*-specific PBMCs and reactive T cell compartment. A**, Interferon (IFN)-γ enzyme-linked immmunosorbent spot (ELISpot) well images of 4 × 10^5^ PBMCs cultured with media, PMA/ionomycin (PMA/ion), or *T. cruzi* lysate (TcLysate/Tc) and 1 × 10^4^ PBMCs stimulated with PMA/ionomycin (PMA/ion (10 000)). Individual dog identification (ID) and results of multiplex serology are denoted above wells. B-C, Numbers of IFNγ-producing PBMCs in the ELISpot assay responding to *T. cruzi* parasite lysate **(B)** or PMA **(C)** adjusted to 1 × 10^6^ or per 1 × 10^4^ PBMCs, respectively. Each square and dot represents an individual dog, while each diamond represents one of three US dog controls to ensure plate reproducibility. Dogs are grouped by multiplex serology negative (Sero -) or serology positive (Sero +) from Argentina or seronegative dogs from United States. Statistical significance was determined by one-way ANOVA using Prism v4.0c (GraphPad). Horizontal line, threshold for positive/negative response.

Measurement of *T. cruzi*-specific T cells has proven useful for confirming and monitoring infection status in humans (23, 27). PBMCs from dogs were evaluated for IFNγ production by ELISpot following stimulation with *T. cruzi* lysate or PMA/ionomycin. Using 20 spot forming units (SFU)/10^6^ PBMCs as a cutoff for positive ELISpot responses, 95% (19/20) of dogs seropositive by multiplex were also positive in the ELISpot assay while 96% (24/25) of multiplex seronegative dogs from Argentina or the United States were ELISpot negative (Figure 2 and Table 1). Significantly higher frequencies of IFNγ-producing T cells in seropositive animals following PBMC incubation with PMA/ionomycin suggested these dogs had a higher overall active T cell compartment as compared to seronegative dogs (Figure 2C). Little to no spontaneous IFNγ production confirms the specificity of the ELISpot assay to identify *T. cruzi*-specific T cells in the dog.

**Figure 3:**
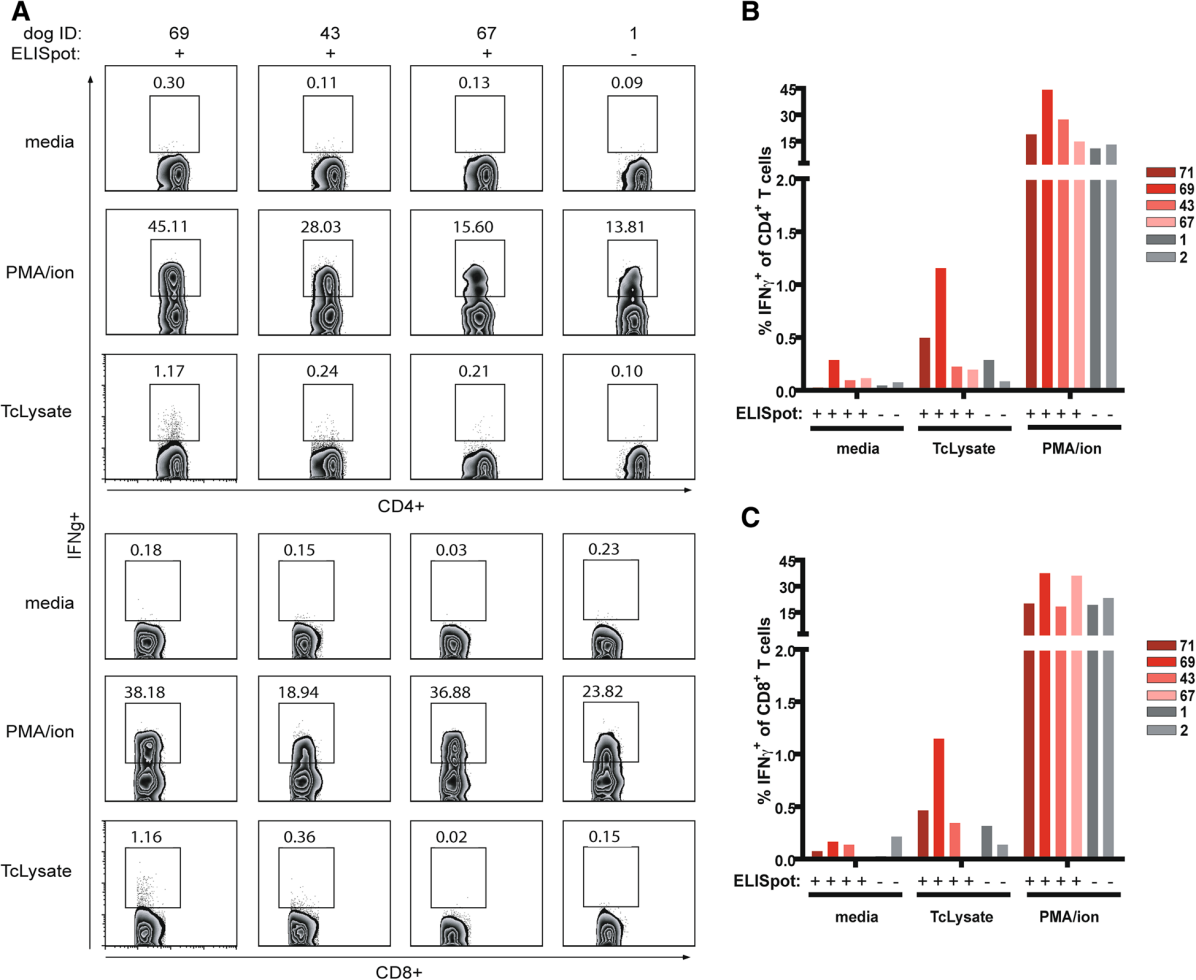
**Intracellular IFNγ staining identifies *T. cruzi*-specific CD4**^**+ **^**and CD8**^**+ **^**T cells. A**, Representative flow plots of intracellular cytokine staining of T cells from ELISpot positive (71, 69, 43, 67) and negative canine (1,2) PBMC cultures incubated with media, PMA/ionomycin, or *T. cruzi* lysate (TcLysate). Numbers indicate percentage of IFNγ-producing CD4^+^ or CD8^+^ T cells. B-C, Percentage of IFNγ-producing CD4^+^**(B)** and CD8^+^**(C)** T cells from individual dogs. Each bar represents a dog from Argentina (red) or United States (gray). Results of reactivity in IFNγ ELISpot assay are denoted below each bar.

To phenotype the IFNγ producing cells, PBMCs from a subset of ELISpot positive and negative dogs were incubated with *T. cruzi* lysate and assessed for intracellular IFNγ production and co-stained for expression of CD4^+^ or CD8^+^ (Figure 3A). Of the four ELISpot positive dogs, three possessed CD4^+^ and CD8^+^ T cells producing IFNγ in response to lysate stimulation, while one dog, dog 67, had only CD4^+^ T cells producing IFNγ in response to lysate stimulation (Figure 3B-C). Taken together, these data demonstrate that a high percentage of *T. cruzi*-infected dogs have measurable CD4^+^ and CD8^+^ T cells responsive to *T. cruzi* lysate.

**Figure 4:**
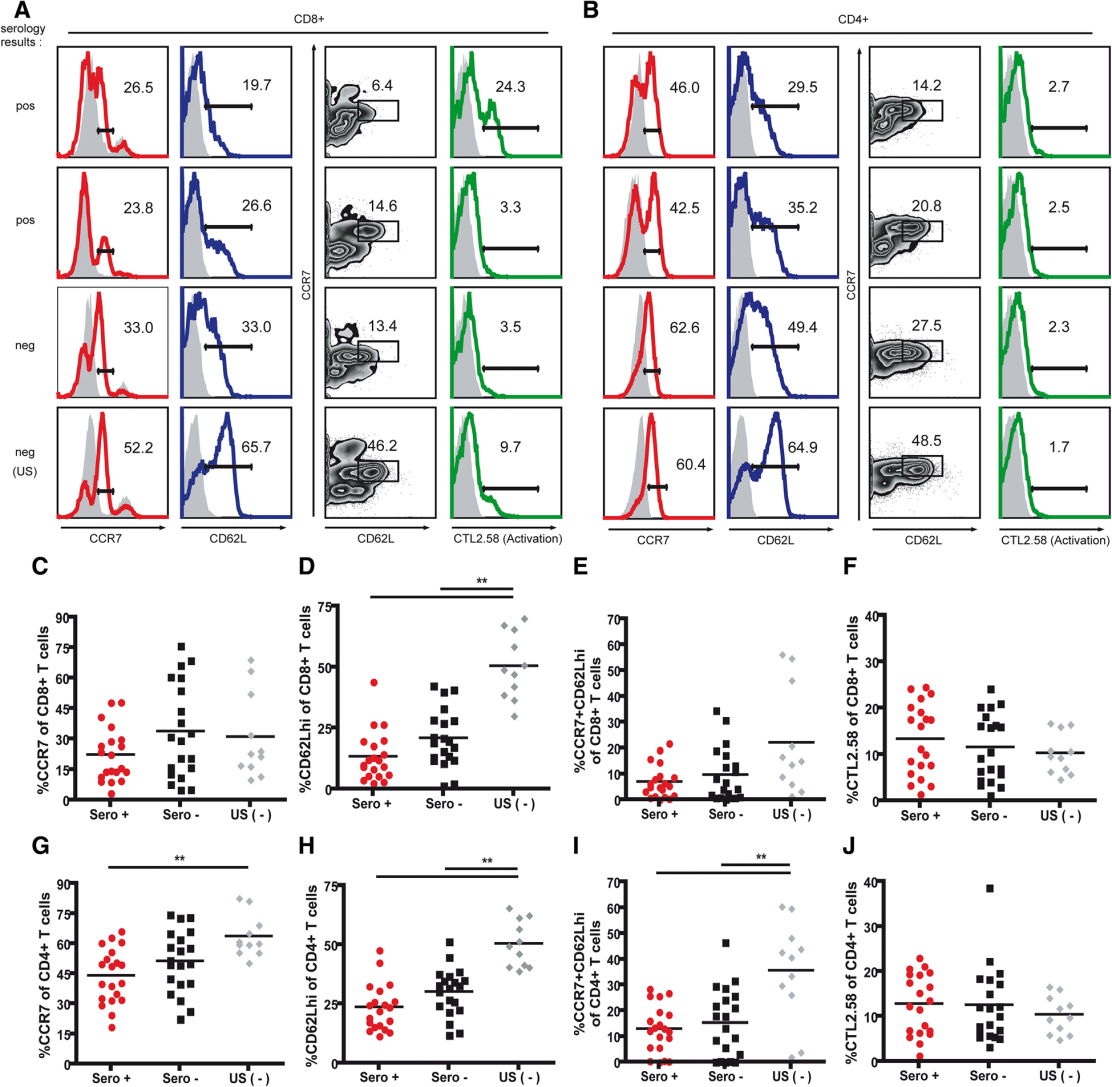
**T cells from seropositive dogs show signs of activation through decreased CCR7 and CD62L expression.** T cells from twenty seropositive and twenty seronegative Argentinian dogs were stained with antibodies for CCR7, CD62L, and CTL2.58, an activation marker. **A-B**. Histograms of CCR7, CD62L, and CTL2.58 expression and dot plots of CCR7 and CD62L co-expression on CD8^+^**(A)** and CD4^+^**(B)** T cells. Numbers indicate percentages of either CD8^+^ or CD4^+^ cells expressing CCR7 **(C, G)**, CD62L **(D, H)**, CCR7 and CD62L **(E, I)**, or the activation marker, CTL2.58 **(F, J)**. Statistical significance was determined by one-way ANOVA using Prism v4.0c (GraphPad). ** designates *p* < 0.001.

As T cell surface markers of activation and effector T cell memory correlate with *T. cruzi* infection and disease severity in humans [26,34], we next evaluated the impact of *T. cruzi* infection on surface antigen expression on canine T cells. Recent validation of T cell surface markers identifying canine T cells with activated (CTL2.58^+^) and naïve or central memory (CCR7^+^CD62L^hi^) phenotypes has provided cell surface markers for delineating canine T cell phenotypes (Additional file 1 and Hartley and Tarleton, unpublished). Examining the CD8^+^ (Figure 4A) and CD4^+^ (Figure 4B) T cell compartments, both T cell subsets expressed measurable levels of CCR7, CD62L, and CTL2.58. The expression and co-expression of naïve or central memory markers CCR7 and CD62L in T cells from healthy US control dogs were consistently higher or significantly higher as compared to the Argentinean dogs for both CD8^+^ and CD4^+^ T cell subsets (Figure 4C-E, G-I). These differentiating results most likely reflect the inherent environmental variation of dogs living in the United States receiving preventative care versus dogs living in a *T. cruzi*-endemic region with limited accessible veterinary care, and thus a higher likelihood of harboring a range of infectious agents. The antibody CTL2.58, a reagent likely recognizing recently activated T cells and which showed strong reactivity with both Con A stimulated canine T cells (Additional file 1), did not appear differentially expressed across any of the dog groups (Figure 4F and J), but high CTL2.58 expression by CD8^+^ T cells from a seropositive dog (Figure 4A) suggested recent T cell activation had occurred in this dog. Overall, these T cell surface markers correlate a positive *T. cruzi* serological status with a robust activated *T. cruzi*-specific T cell response.

## Discussion

*T. cruzi*-infected dogs are a major factor in transmission of *T. cruzi* to humans [3] and are present in both rural and urban settings [35]. Thus, identification of infected dogs is critical for design and appropriate targeting of *T. cruzi* transmission control and intervention strategies. Towards this goal, we first tested the ability of an improved serological assay utilizing *T. cruzi* proteins expressed in mammalian-stage parasites [20] to detect *T. cruzi* infection in dogs. Though the protein panel selected for this multiplex assay was originally defined for discriminating human sera, initial tests with a subset of dog sera or plasma revealed high reactivity as well (Figure 1). In particular, a six-protein subset was highly discriminatory for serological status, with a minimum two-protein reactivity of four standard deviations above seronegative animals identifying 94% (17/18) of multiplex serology positive dogs that were positive by conventional serology (Table 1). Selection and screening of additional proteins with canine plasma samples, including a broader set of discordant dogs, would likely improve upon this multiplex bead array assay. A single test format that conclusively determines infection status, as opposed to the current method of using multiple platforms and the resulting “discordant” samples, would represent an improvement in diagnostics for dogs.

To our knowledge, this was the first study to evaluate and correlate the *T. cruzi*-specific antibody response with T cell phenotype and effector cytokine production in dogs. In humans, decreases in antibody responses and in the frequency of *T. cruzi*-induced IFNγ-production by T cells and following benznidazole treatment have provided methods to evaluate infection and treatment efficacy in people. In the present study, our aim was to determine if antibody responses in dogs naturally infected with *T. cruzi* correlated with *T. cruzi*-specific T cell responses, in efforts for ultimately identifying methods to track canine *T. cruzi* infection. In contrast with human IFNγ-ELISpot assays, where IFNγ-producing PBMCs were detected in 58% of seropositive and 35% of conventional seronegative individuals living in areas of active transmission [36], 95% of seropositive dogs and 4% of seronegative dogs were positive in the canine IFNγ-ELISpot assay. These data suggest dogs not only develop robust T cells response during *T. cruzi* infection but the *T. cruzi*-specific immune responses are much more apparent than those present in humans. Relatively shorter terms of *T. cruzi* infection in dogs, i.e. months to years versus decades in humans, and the resultant decreased potential for T cell exhaustion, as observed in human patients [34], may explain the disparity in seroreactivity and ELISpot results observed between humans and dogs. Intracellular cytokine staining allowed us to identify both CD4^+^ and CD8^+^ T cells as responsible for IFNγ production in selected dogs (Figure 3) at various, but low frequencies. Similar low frequency of IFNγ response to *T. cruzi* lysate has been documented in human Chagasic patients [27]. The robust frequency of IFNγ-producing T cells observed in seropositive dogs in the ELISpot assay provides a diagnostic method to detect, monitor, and track canine *T. cruzi* infection. We would predict that, as in humans, dogs treated with curative anti-*T. cruzi* drugs would exhibit predictable changes in the frequencies of these *T. cruzi*-specific T cell responses [23].

Additionally, the correlation of various T cell surface markers with drug-induced cure has defined T cell biomarkers for assessing antigen encounter and parasite burden during murine *T. cruzi* infection [30]. Similar work in humans have correlated T cell surface marker changes with post benznidazole treatment periods [23], although the inability to measure these markers on bona fide *T. cruzi*-specific T cells [27] provides a less dependable marker of treatment efficacy than is possible in the mouse, where these T cells area easily monitored using MHC tetramers. Utilizing T cell-specific surface markers validated for the dog, phenotyping of canine PBMCs provided a method to assess hypothesized differences in global T cell surface marker expression between seropositive and seronegative animals. Though not statistically significant, higher expression of CCR7 and CD62L in seronegative versus seropositive dog T cells are consistent with these cell surface markers identifying naïve or central memory T cells. Seropositive dogs, on the other hand, possessed CD4^+^ and CD8^+^ T cells expressing decreased levels of CCR7 and CD62L. Further monitoring of these T cell surface markers during the course of canine *T. cruzi* infection and particularly on IFNγ-producing cells, would greatly enhance our understanding development, maintenance, and persistence of the canine T cell immune responses for evaluating various intervention strategies focused on the dog.

In addition to being important players in the transmission of *T. cruzi* to humans [3,4,6,7], as natural hosts that suffer clinical manifestations of Chagasic disease [37,38], and as models for testing of anti-trypanosomal drugs [39], canines are strong translational models for studying human *T. cruzi* infection. Understanding and characterizing the canine *T. cruzi*-specific immune response is imperative for identifying infected canines in regions of active transmission, evaluating the efficacy of targeted canine intervention strategies, including potential vaccine strategies, and overall improving our knowledge of antibody and T cells responses during canine disease. The full impact and application of serology and T cell responses for tracking and monitoring canine *T. cruzi* infection and evaluating *T. cruzi* transmission dynamics have yet to be determined.

## Competing interests

The authors declare they have no competing interests.

## Authors’ contributions

ANH and RLT conceived and designed the experiments. ANH, GC, MMO, and SG performed the experiments. ANH, GC, MMO, and RLT analyzed the data. ANH, GC, MMO, and RLT contributed reagents/materials/analysis tools. ANH and RLT wrote the manuscript. All authors read and approved the final manuscript.

## Supplementary Material

Additional file 1 **Induction of CTL2.58 expression on canine PBMCs following stimulation with Con A.** Canine PBMCs incubated two days with media or ConA, harvested, and stained to identify CD4^+^ and CD8^+^ T cells and the expression of CTL2.58 (conjugated to PeCy7). Numbers indicate the percentage of CD4^+^ and CD8^+^ T cells among PBMCs (left column) and the percentage of these subpopulations positive for the activation molecule recognized by CTL2.58 antibody with or without Con A stimulation (center and right columns). Click here for file
