# Supplementary figures and images for: Frequency of IFNγ-producing T cells correlates with seroreactivity and activated T cells during canine Trypanosoma cruzi infection
Source: Vet Res. 2014 Jan 23;45(1):6. doi: 10.1186/1297-9716-45-6 (PMC3907784; doi:10.1186/1297-9716-45-6)

**
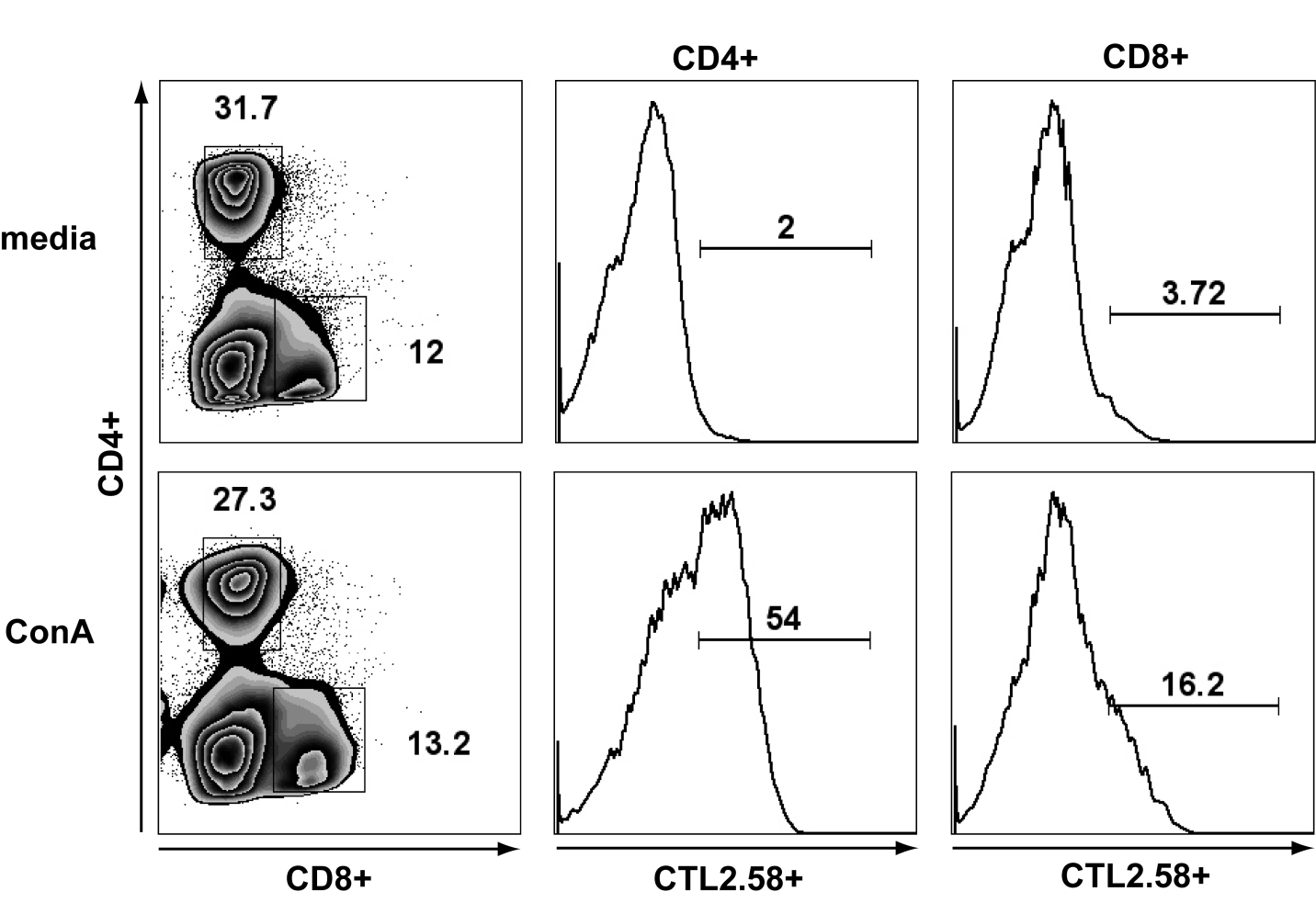
**

Supplement: Additional file 1 — Induction of CTL2.58 expression on canine PBMCs following stimulation with Con A. Canine PBMCs incubated two days with media or ConA, harvested, and stained to identify CD4+ and CD8+ T cells and the expression of CTL2.58 (conjugated to PeCy7). Numbers indicate the percentage of CD4+ and CD8+ T cells among PBMCs (left column) and the percentage of these subpopulations positive for the activation molecule recognized by CTL2.58 antibody with or without Con A stimulation (center and right columns). [file 1297-9716-45-6-S1.docx]
